# Analyses of association between PPAR gamma and EPHX1 polymorphisms and susceptibility to COPD in a Hungarian cohort, a case-control study

**DOI:** 10.1186/1471-2350-11-152

**Published:** 2010-11-02

**Authors:** Andras Penyige, Szilard Poliska, Eszter Csanky, Beata Scholtz, Balazs Dezso, Ivan Schmelczer, Iain Kilty, Laszlo Takacs, Laszlo Nagy

**Affiliations:** 1 Department of Human Genetics, University of Debrecen, Debrecen, Hungary; 2 Department of Biochemistry and Molecular Biology, Research Center for Molecular Medicine, University of Debrecen, Debrecen, Hungary; 3 Clinical Genomics Center, Medical and Health Science Center, Research Center for Molecular Medicine, University of Debrecen, Debrecen, Hungary; 4 Apoptosis and Genomics Research Group of the Hungarian Academy of Sciences, Research Center for Molecular Medicine, University of Debrecen, Debrecen, Hungary; 5 Department of Pulmonology, Medical and Health Science Center, University of Debrecen, Debrecen, Hungary; 6 Department of Pathology, University of Debrecen, Medical and Health Science Center, Debrecen, Hungary; 7 Pfizer Global Research and Development, Sandwich, UK; 8 Biosystems International SAS, Evry, France; 9 Department of Pulmonology, Semmelweis Health Care Center of Miskolc, Miskolc, Hungary

## Abstract

**Background:**

In addition to smoking, genetic predisposition is believed to play a major role in the pathogenesis of chronic obstructive pulmonary disease (COPD). Genetic association studies of new candidate genes in COPD may lead to improved understanding of the pathogenesis of the disease.

**Methods:**

Two proposed casual single nucleotide polymorphisms (SNP) *(rs1051740, rs2234922) *in microsomal epoxide hydrolase (*EPHX1*) and three SNPs *(rs1801282, rs1800571, rs3856806) *in peroxisome proliferator-activated receptor gamma (*PPARG*), a new candidate gene, were genotyped in a case-control study (272 COPD patients and 301 controls subjects) in Hungary. Allele frequencies and genotype distributions were compared between the two cohorts and trend test was also used to evaluate association between SNPs and COPD. To estimate the strength of association, odds ratios (OR) (with 95% CI) were calculated and potential confounding variables were tested in logistic regression analysis. Association between haplotypes and COPD outcome was also assessed.

**Results:**

The distribution of imputed *EPHX1 *phenotypes was significantly different between the COPD and the control group (P = 0.041), OR for the slow activity phenotype was 1.639 (95% CI = 1.08- 2.49; P = 0.021) in our study. In logistic regression analysis adjusted for both variants, also age and pack-year, the rare allele of His447His of *PPARG *showed significant association with COPD outcome (OR = 1.853, 95% CI = 1.09-3.14, P = 0.0218). In haplotype analysis the GC haplotype of *PPARG *(OR = 0.512, 95% CI = 0.27-0.96, P = 0.035) conferred reduced risk for COPD.

**Conclusions:**

The "slow" activity-associated genotypes of *EPHX1 *were associated with increased risk of COPD. The minor His447His allele of *PPARG *significantly increased; and the haplotype containing the minor Pro12Ala and the major His447His polymorphisms of *PPARG *decreased the risk of COPD.

## Background

Chronic obstructive pulmonary disease (COPD) is an increasing and serious public health problem representing the fourth leading cause of death globally. COPD is a complex human disease, associated with persistent airway inflammation, protease-anti-protease imbalance, oxidative stress, chronic obstructive bronchitis and emphysema, resulting in progressive airflow limitation that is not substantially reversed by bronchodilators. Importantly, it is a smoking-related disorder and cigarette smoking is the major environmental risk factor for development of COPD. However, the fact that only a subset of smokers (15-20%) develops clinically significant symptoms suggests that genetic predisposition also plays role in the development of COPD [1,2].

Previous genetic association and genome-wide linkage studies have identified several candidate genes that might be involved in the pathogenesis of COPD [3-7].

In our case-control study, five putative causal single nucleotide polymorphisms (SNPs) in two genes - microsomal epoxide hydrolase (*EPHX1*) and peroxisome proliferator-activated receptor gamma (*PPARG*) - were chosen to analyze their association with COPD.

*PPARG *is a member of nuclear hormone receptors, implicated in adipocyte differentiation and involved in macrophage activation and dendritic cell biology [8]. An anti-inflammatory role of *PPARG *was also reported based on its inhibitory effect on pro-inflammatory transcription factors such as NF-κB and AP-1 [9,10]. The NCBI SNP database features more than 700 polymorphisms of PPARG, many of them intronic or synonymous variant and generally lack of information regarding population diversity.

Several studies on the polymorphisms of *PPARG *such as Pro12Ala and His447His were previously performed in relation to inflammatory diseases such as IBD, type 2 diabetes and recently it has been suggested that *PPARG *polymorphisms were associated with the risk of asthma [11]. Since *PPARG *might be involved in the regulation of pro-inflammatory signaling pathways and it is generally accepted that COPD is associated with an abnormal inflammatory response, genetic polymorphisms in *PPARG *could be implicated in the susceptibility to COPD risk [12]. That may provide a theoretical basis for the study of this new candidate gene in COPD development. Consequently, we examined the association between three SNP polymorphisms of *PPARG *gene and COPD.

*EPHX1 *is an enzyme associated with the metabolism and detoxification of xenobiotic chemicals; it plays an important role in the general oxidative defense of lung. Several polymorphisms are known in *EPHX1 *including two relatively common SNPs, the exon 3 Tyr113His (*rs1051740*) and exon 4 His139Arg (*rs2234922*) variants. These two variant alleles have been suggested to be associated with altered *EPHX1 *enzyme activity [13]. Substitution of Tyr113 for His decreases *EPHX1 *activity (slow allele), whereas substitution of His139 for Arg increases *EPHX1 *activity (fast allele). It was found that the slow metabolizing form of *EPHX1 *was associated with an increased risk for COPD and evidence supporting this association has been replicated in several case-control genetic association studies [7,14,15]. However, the evidence supporting this association has not been consistent, several genetic association studies failed to show association between these polymorphisms and COPD [16,17]. In this study we have chosen the Tyr113His and His139Arg polymorphisms to validate our Hungarian population for COPD association studies.

All together a total of five SNPs in two candidate genes were genotyped in order to examine their association with COPD susceptibility by using Hungarian cohorts, a previously uninvestigated population with the highest COPD mortality rate among men in Europe [18].

## Methods

### Study populations

The study is a case-control genetic association study in a Central-European Caucasian population. In our analysis the cases included 272 and the control subjects included 301 age-matched Hungarian individuals. The recruitment and the clinical analyses of patients were conducted at the Department of Pulmonology, Medical and Health Science Center, University of Debrecen, according to the Global Initiative for Chronic Obstructive Lung Disease (GOLD) criteria. The Research Ethics Committee of University of Debrecen Medical and Health Science Center approved the clinical protocol and the study. Written informed consent was obtained before the subjects entered the study. The investigator explained the nature, purpose and risk of the study and provided the subject with a copy of the information sheet. The subjects were then given time to consider the study's implication before deciding to participate.

Before starting sample collection, we defined inclusion and exclusion criteria of diseased and healthy patients. Inclusion criteria for COPD subjects were age 40 to 65 years old. Patients must have predicted value of forced expiratory volume at 1 second (FEV1) 50%-80% and FEV1/FVC% <70% (stage 2 according to GOLD criteria). Inclusion criteria for control patients were age between 40 and 65 years; normal spirometry, FEV1 ≥ 90% (predicted value) and FEV1/FVC% ≥ 80%. All patients must be current or ex-smoker (minimum 15 pack years).

Exclusion criteria for all patients: plasma IgE level >70 U/ml, alpha1-antripsin deficiency, evidence of asthma, atopic disease, history of lung disorders, respiratory infection in the past 3 months, other inflammatory diseases (e.g. inflammatory bowel disease, rheumatoid arthritis, psoriasis etc.), autoimmune diseases (lupus, sclerosis), cancer, positive plasma test for HIV, Hepatitis B or Hepatitis C.

### Genotyping

Genomic DNA was extracted from peripheral blood using E.Z.N.A. Blood DNA Midi Kit (Peqlab Biotechnologie) according to the manufacturer's protocol. Quality of the DNA samples was checked by agarose gel-electrophoresis and quantitated by NanoDrop1000. All SNPs were genotyped using TaqMan genotyping assays (Additional File 1, Table S1). Samples were measured in duplicates and nuclease-free water was used as no-template control. Following PCR amplification the end-point fluorescence was read with the ABI 7900 HT instrument and genotypes were assigned using SDS software (Applied Biosystems). The average genotyping success rate of at least 95% was attained for each SNP.

### Alveolar macrophage (AM) and peripheral blood monocyte (MO) collection

Bronchoalveolar lavage fluid (BALF) samples were collected by fiber-optic bronchoscopy from healthy and COPD patients. AMs were separated by Percoll (Amersham Biosciences) gradient centrifugation. Total cell number was determined by counting in hemocytometer. Differential cell count was assessed on hematoxylin-eosin stained cytospin slides before and after the gradient separation. Over 95% AM purity was reached after separation.

50 ml heparin treated venous blood was collected from healthy and diseased patients. MOs were separated by Ficoll gradient centrifugation using anti-CD14 conjugated microbeads (>98% MO) (VarioMACS, Miltenyi Biotec.). 5-5 control and COPD patients were recruited in this experiment.

### TaqMan RT-QPCR

Total RNA was isolated from peripheral blood monocytes, alveolar macrophages, lung tissue and adipose tissue using Trizol reagent (Invitrogen). First strand cDNA was generated from 5 ug total RNA using cDNA Archive Kit (Applied Biosystems). For RT-QPCR reaction 200 ng cDNA/sample and 2X TaqMan PCR mix (Applied Biosystems) was used. Reactions were run in ABI Prism HT 7900 instrument. We have designed primer pair and probe for measuring *PPARG *mRNA level. Forward primer: 5'GATGACAGCGACTTGGCAA, reverse primer: 5'CTTCAATGGGCTTCACATTCA, probe: 5'FAM-CAAACCTGGGCGGTCTCCACTGAG-3'TAMRA. The housekeeping gene cyclophilin A was used as a normalizer gene: forward primer: 5'ACGGCGAGCCCTTGG, reverse primer: 5' TTTCTGCTGTCTTTGGGACCT, probe: 5'FAM-CGCGTCTCCTTTGAGCTGTTTGCA-3'TAMRA. Relative gene expression levels were calculated by comparative Ct method. Statistical analysis was performed in GraphPad Prism using non-parametric test (Mann-Whitney U-test).

### Immunohistochemistry

Tissues for morphology and immunostainings were obtained from the files of the Pathology Department of University of Debrecen and were freshly fixed in 10% neutral formalin and embedded in paraffin followed by hematoxyline and eosine (HE) staining using standard methods. Immunohistochemistry (IHC) for alveolar macrophages (AM) was carried out using PPARG, CD68 and DCSign (Santa Cruz) monoclonal antibodies by means of immunoperoxidase staining as described earlier [19,20]. Double immunofluorescence staining was performed as described earlier [21] using CSAII detection kit with FITC-labeled tyramine followed by an immunofluorescent staining for DCSign with streptavidin-texas red fluorochrome. Nuclear counterstaining was made with DAPI (blue fluorescence).

### Statistical analysis

Differences between cases and the control group concerning demographic and main clinical data were analyzed by Mann-Whitney *U*-test and Pearson χ^2 ^test. Genotype data for each SNP were tested for departures from Hardy-Weinberg equilibrium (HWE) separately in case and control populations using a goodness-of-fit χ^2^-test or the exact test to estimate P values. HWE calculations were done by using the HWE tool http://ihg.gsf.de/cgi-bin/hw/hwa1.pl. The significance of differences in genotype and allele frequencies between patients and controls were tested by using either χ^2 ^analyses or Fisher's exact test where appropriate [22]. To assess the degree of association between each of the SNPs and COPD odds ratios with 95% confidence intervals (OR, 95% CI) were calculated using logistic regression analysis; the model was adjusted for SNPs, pack-year, age and gender. All single locus association tests were performed using the STATA 9.0 statistical package (except where otherwise stated).

Haplotype frequencies were estimated for control and patient groups separately with the Full-Precise-Iteration algorithm implemented in the SHEsis software http://analysis.bio-x.cn/myAnalysis.php. The extent of linkage disequilibrium between pairs of biallelic markers was determined using both the standardized disequilibrium and correlation coefficients (given as Lewontin's D' and r^2, ^respectively) and association between haplotypes and COPD was assessed by the χ^2^-test or the exact test as implemented in the program SHEesis [23,24]. Correction for multiple testing was not used in the analysis of the association genotype and allele frequencies because: (i) the EPHX1 polymorphisms were known to be functional and (ii) the gene is considered a susceptibility gene for COPD; and (iii) in case of the PPARG polymorphisms the studied individual alleles were not independent. *A posteriori *estimates of study power were assessed by means of Quanto software http://hydra.usc.edu. We have estimated the power of our study with the following parameters: sample size of 272 cases; control/case ratio of 1.1; minor allele frequencies (MAF) are in the range from 0.12 to 0.3; log-additive model; disease prevalence of COPD 5%. Assuming these parameters our study had ~50% power to detect a genotype relative risk (GRR) of 1.4 for MAF = 0.12, or ~79% power to detect a GRR of 1.6 for the same MAF; while it had ~76% power to detect a GRR of 1.4, and ~96% power to detect a GRR of 1.6 for MAF = 0.3 at an α = 0.05 significance level.

## Results

Of the 573 subjects genotyped, 61.8% of the subjects were men. The proportion of males was higher among cases (69.85% to 54.48%) but there is no significant difference in the mean age of controls and cases. Cases had been exposed to more tobacco smoke as evidenced by the difference in pack-years but the difference is not significant, COPD patients had a much larger reduction in lung function, typical for a clinical COPD population (Table 1).

**Table 1:** Clinical features of the study population

Parameter	Cases (N = 272)	Controls (N = 301)	P value
Male (%)	190 (69.85)	164 (54.48)	0.781

Age (±SD)*	63.87 (±8.96)	64.29 (±9.07)	0.577

Pack-Years (±SD)*	38.75 (±19.91)	34.76 (±14.71)	0.120

FEV1% predicted (±SD)*	47.17 (±13.69)	99.28 (±9.76)	<0.0001

FEV1/FVC% predicted (±SD) *	57.57 (±10.12)	87.10 (±35.24)	<0.001

Data presented as mean ± SD. FEV1: forced expiratory volume in one second, FVC: forced vital capacity, *Mann-Whitney U-test was used.

All genotype frequencies were consistent with Hardy-Weinberg equilibrium for both SNPs of the *EPHX1 *gene, and the genotype and allele frequencies did not differ significantly between cases and the control group (Table 2). The assessment of the association of individual SNPs with COPD showed that homozygosity for the minor allele increased the risk of disease in case of Tyr113His polymorphism ("slow" allele) (OR = 1.345; 95% CI = 0.96 - 1.91; P = 0.095) reduced it in case of the His139Arg SNP ("fast" allele) (OR = 0.675; 95% CI = 0.27 - 1.69; P = 0.399). However, none of these SNPs were significantly associated with COPD even after adjusting the model for gender, age and pack-years in logistic regression.

**Table 2:** Allele and genotype frequencies of examined EPHX1 gene polymorphisms

Gene Symbol	SNP ID	Allele frequency		Genotype frequency			^§^Hardy-Weinberg Equilibrium	OR (95% CI)
	rs1051740 (Tyr113His)	T	C	TT (%)	TC (%)	CC (%)	P value	

Controls		0.723	0.277	154 (53.3)	110 (38.1	25 (8.7)	0.401	1.11 (0.86-1.44)
Cases		0.701	0.299	127 (47.4)	122 (45.5)	19 (7.1)	0.154	

	rs2234922 (His139Arg)	A	G	AA (%)	AG (%)	GG (%)		

Controls		0.779	0.221	171 (60.0)	102 (35.8)	12 (4.2)	0.507	0.88 (0.66-1.18)
Cases		0.799	0.201	169 (62.8)	92 (64.2)	8 (2.9)	0.280	

^§^χ^2^-test was used, OR: odds ratio, CI: confidence interval

Frequencies for the four SNP based haplotypes were estimated for cases and controls. Although the "slow activity" CA (His^113^-His^139^) haplotype was more frequent among cases (24.8% versus the 20.8% in controls), the overall distribution did not differ significantly between the two groups (P = 0.736). Furthermore, alleles of the two loci in *EPHX1 *are in complete linkage equilibrium as shown by the pair-wise standardized disequilibrium coefficient (D' = 0.036).

Due to the presence of these coding variants, marked variations in *EPHX1 *activity have been reported previously. Therefore we have assessed the association of the predicted (rapid", "normal", "slow" and "very slow") *EPHX1 *phenotypes with the development of COPD [25,26]. The distribution of predicted *EPHX1 *activity was significantly different between control subjects and COPD patients (P = 0.041). In the analysis of predicted phenotypes the COPD group had higher proportion of the predicted "slow" phenotype. Consequently the slow phenotype significantly raises the risk of developing COPD [OR = 1.639; 95% CI = 1.06-2.49; P = 0.021)] in our case control study (Table 3).

**Table 3:** The distribution of the predicted EPHX1 phenotypes

Predicted EPHX1 activity	Controls n (%)	Cases n (%)	^§^P value	Contingency tables OR (95% CI)	P value
Normal	144 (53.1)	123 (48.4)		1 (reference)	
		
Slow	55 (20.2)	77 (30.3)		1.64 (1.08-2.49)	0.021
		
Very slow	17 (6.2)	9 (3.5)	0.041	0.62 (0.27-1.44)	0.306
		
Rapid	55 (20.2)	45 (17.7)		0.96 (0.59-3.19)	0.855

Normal: exon3 Tyr/Tyr and exon 4 His/His or exon 3 Tyr/His and exon 4 His/Arg; Slow: exon 3 Tyr/Tyr and exon 4 His/His; Very slow: exon 3 His/His and exon 4 His/His; Rapid: exon 3 tyr/Tyr and exon 4 Arg/Arg or His/Arg

^§^χ^2^-test was used for the distribution of predicted phenotypes, OR: odds ratio, CI: confidence interval

**Figure 1:**
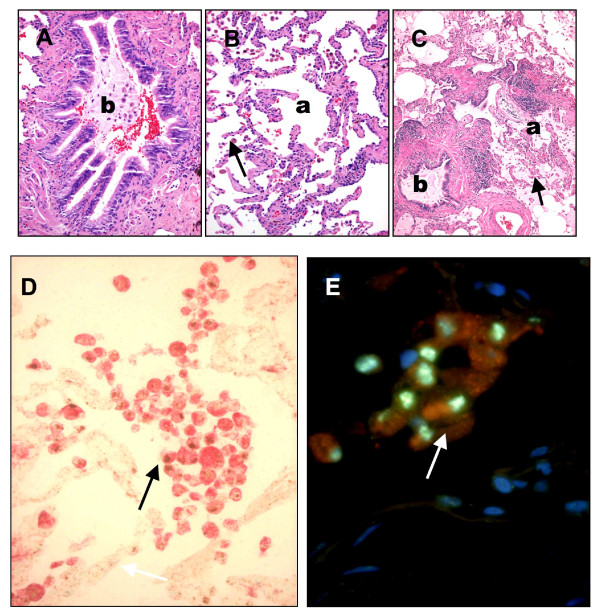
**Morphology and immunohistochemistry of COPD-associated lung lesions**. **A**: Chronic bronchitis. **B**: Centriacinar emphysema. **C**: Advanced active bronchitis with fibrosis and emphysema. ***A-C***, hematoxylin-eosin staining; **D**: CD68-PPARG coexpression with double IHC staining using alkaline phosphatase [red cytoplasm-CD68] and diamino-benzidine [brown nuclei-PPARG]. **E**: Cells with red fluorescence and green nuclei DCSign-PPARG double fluorescence staining. Nuclear counter-staining is DAPI. *Indications*: **b**, bronchus; **a**, alveolar spaces; **arrows**, alveolar macrophages. *Original magnifications: ***A-D **20×; **E **40×;

**Figure 2:**
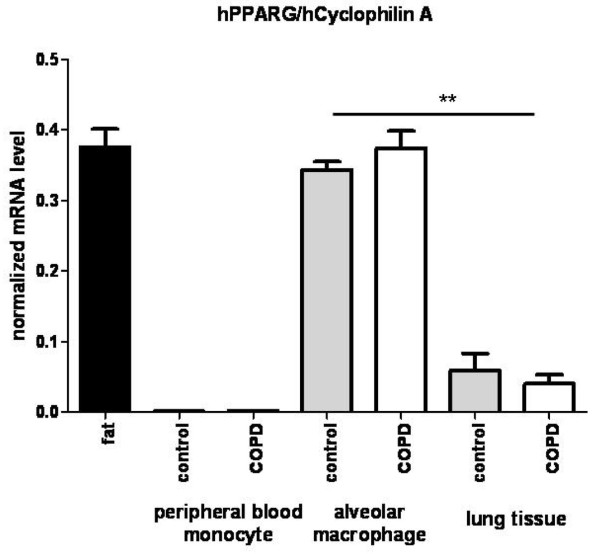
**mRNA expression of PPARG**. PPARG showed as high as mRNA expression level in alveolar macrophages as in subcutan fat and also showed expression in the whole lung tissue with significantly lower level (Mann-Whitney U test). However it was not expressed in peripheral blood monocytes. Data presented normalized values of RT-QPCR measurements; Cyclophilin A was used as housekeeping gene. Grey bars represent mean values of 5 control patients; white bars represent mean values of 5 COPD patients. ** p < 0.01.

High expression level of *PPARG *mRNA in the lung has been reported previously [27,28]. We examined *PPARG *expression at protein level in surgical lung tissue samples. *PPARG *protein is expressed in the lung and most of the *PPARG *protein derived signals co-localized with the expression of a typical macrophage marker CD68 and the dendritic cell marker DCSign (Figure 1). *PPARG *expression was also measured at mRNA level. mRNA expression is enriched in AM relative to total lung tissue, however we did not find differences in expression level between COPD and healthy individuals (Figure 2). Thus, genetic variants, rather than the expression of the *PPARG *gene could be associated with the development of COPD.

We have genotyped three exonic SNPs (*rs10801282 (Pro12Ala)*, *rs3856806 (His447His) *and *rs1800571 (Pro113Gln)*) in *PPARG *gene, but the *rs1800571 *locus was left out from the analysis because it was homozygous for the major C allele in all individuals. The other two SNPs were in HWE and showed linkage disequilibrium, the extent of LD between *rs10801282 *and *rs3856806 *was found to be *D' *= 0.673, although the pair showed lower LD with respect to their correlation coefficient (*r*^*2 *^= 0.42).

The single loci allelic and genotypic analysis found no significant association between the two coding variants of *PPARG *and COPD. In logistic regression applying a model adjusted for both SNPs, age and pack-years, the rare variant of His447His polymorphism was significantly associated with increased odds for COPD (OR = 1.853, 95% CI = 1.09 - 3.14, P = 0.021). The minor Ala allele of the Pro12Ala variant had an OR of 0.679, suggesting a protective effect, however it did not reach significance (95% CI = 0.40 - 1.14, P = 0.145) (Table 4).

**Table 4:** Allele and genotype frequencies of examined PPARG gene polymorphisms

Gene Symbol	SNP ID	Allele frequency		Genotype frequency			^§^Hardy-Weinberg Equilibrium	Logistic Analysis	
	rs1801282 (Pro12Ala)	C	G	CC (%)	CG (%)	GG (%)	P value	OR (95% CI)	^#^P value

Controls		0.864	0.136	217 (75.2)	64 (22.4)	7 (2.4)	0.398	0.68 (0.40-1.14)	0.15
Cases		0.874	0.126	199 (76.2)	67 (22.3)	4 (1.5)	0.869		

	rs3856806 (His447His)	C	T	CC (%)	CT (%)	TT (%)	P value	OR (95% CI)	^#^P value

Controls		0.882	0.118	224 (78.8)	53 (18.7)	8 (2.5)	0.09	1.85 (1.09-3.14)	0.02
Cases		0.862	0.138	199 (74.0)	65 (24.5)	5 (1.5)	0.57		

^§^χ^2^-test was used, OR: odds ratio, CI: confidence interval

^#^Adjusted for age and pack-year

We have estimated the frequency of the possible two-SNP haplotypes of *PPARG *and assessed the association between haplotypes and COPD development. There was a significant difference in the frequency of the GC haplotype involving the rare G variant of the Pro12Ala locus between the two groups (P = 0.035). The strength of association was also assessed, for this haplotype (OR = 0.512; 95% CI = 0.27-0.96). This finding suggests a protective effect for the GC (12Ala/447His) haplotype of the *PPARG *gene for COPD outcome (Table 5).

**Table 5:** The distribution and strength of association of PPARG haplotypes

Haplotypes	COPD (freq.)	Control (freq.)	^§^P value	OR (95% CI)
CC	0.834	0.832	0.914	1.02 (0.74-1.40)

CT	0.039	0.030	0.424	1.31 (0.68-2.50)

GC	0.028	0.053	0.035	0.51 (0.27-0.96)

GT	0.098	0.085	0.429	1.18 (0.78-1.77)

^§^χ^2^-test was used, OR: odds ratio, CI: confidence interval

## Discussion

The aims of this study were to investigate association of *EPHX1 *polymorphisms to COPD in a Hungarian population and to assess possible association between SNPs of *PPARG*, a new candidate gene, and COPD outcome.

In our study of individual SNPs in *EPHX1 *(the exon-3 Tyr113His and exon-4 His139Arg variants), the homozygous minor Thr113His variant showed only a borderline association with the COPD phenotype (P = 0.095), however the level of association was further reduced when the model was adjusted for age, sex and pack-years.

There is complete linkage equilibrium between Tyr113His and His139Arg SNPs and no statistical significance was found in haplotype frequency distribution and their association with COPD phenotype.

Since *EPHX1 *is involved in the detoxification of epoxide intermediates in tobacco smoke, the rate of conversion of these highly reactive compounds could affect an individual's ability to cope with the toxic effect of cigarette smoke [29]. We have reconstructed the frequency of predicted *EPHX1 *activity in patient and control groups [25,26]. The difference in distribution of predicted phenotypes was significant between the two groups. The COPD group had a higher proportion of predicted slow and lower proportion of predicted normal and rapid *EPHX1 *activity. Interestingly the control group showed an excess of very slow phenotypes, but its frequency was low in both groups. In our analysis the slow activity variant of *EPHX1 *enzyme was associated with a significantly increased the risk for COPD. This result provides additional support to the notion that EPHX1 is likely to be involved in COPD pathogenesis.

Previous studies about the potent anti-inflammatory properties of the *PPARG *agonists suggest the use of *PPARG *ligands in COPD therapy [12,30,31] and association of *PPARG *gene polymorphisms with the development of asthma has been reported [11]. These lines of evidence prompted us to investigate *PPARG *gene association existed between the presence of certain *PPARG *gene polymorphisms and COPD outcome. Using IHC staining and RT-QPCR measurements, we have confirmed *PPARG *mRNA and protein expression in lung tissues and particular in AM [32]. Although gene expression level is comparable in patients and healthy individuals, polymorphisms of the gene still could be potential candidate markers of the disease if a functionally altered *PPARG *has a role in the development in COPD.

Among the three SNPs we have genotyped in *PPARG *- rs1801282, rs3856806 and rs1800571 - the rs1800571 was excluded since all individuals carried an identical homozygous genotype [33]. Our single-marker tests for the other two coding variants yielded a significant association for the minor allele of His447His polymorphism in logistic regression adjusted for both SNPs, age, sex and pack-years (OR = 1.853; 95% CI = 1.09-3.14; P = 0.02). The His447His variant did not cause amino acid change and it has no known function. However, several papers pointed out that exonic synonymous SNPs can affect mRNA splicing or stability [34,35]. Any change in these processes could exert a significant effect on protein function, therefore there was a reason to investigate the association of this SNP with COPD. Of course the possibility, that the His447His SNP is tightly linked with an unknown functional variant that determine COPD susceptibility can not be excluded.

A modest pair-wise LD was found between rs1801282 and rs3856806. Since the use of SNP-based haplotypes in genetic association studies may offer a more powerful approach than the use of individual SNPs, a haplotype analysis was also performed. A significant difference was found in the frequency of GC haplotype (containing the minor G allele of Pro12Ala and major C for His447His variant) between the control and COPD groups and the association of this haplotype to COPD outcome was also determined. The GC haplotype confers a significant lower risk for COPD, pointing to a potential functional protective effect of this haplotype.

Interestingly the minor allele of Pro12Ala polymorphism that lowers the binding affinity of *PPARG *protein to peroxisome proliferator response element was found to be associated with lower body mass index, improved insulin sensitivity, decreased risk of type 2 diabetes [36,37] and reduced risk to develop colorectal cancer [38]. Higher frequency of T allele of the His447His C/T polymorphism was observed in colon cancer [39,40].

We are aware of the fact that significant results could prove to be false positives, and a clear limitation of our study is the relatively low sample size. The study has other limitations such as that population stratification should be investigated in these kinds of studies and we did not analyze a second cohort to replicate our results. Possible gene-gene and gene-environment interactions pose a difficulty for genetic analysis of COPD association studies, too. Further studies using larger populations are needed and other variants in the *PPARG *gene should be investigated in order to clarify the association of *PPARG *and individual susceptibility to the development of COPD.

## Conclusions

In summary, our study provided support for the suggested causative role of *EPHX1 *polymorphisms and phenotypes imputed from exon 3 and exon 4 genotype data in COPD outcome in a Hungarian population.

We have carried out the first investigation of *PPARG *gene polymorphisms in a case-control COPD study and characterized the association between individual SNPs and haplotypes in *PPARG *and susceptibility to COPD. Although the GC haplotype has a modest protective effect, it might point toward the potential importance of common alleles with weak effect in heterogeneous diseases, like COPD. The documentation of *PPARG *haplotype association with COPD identifies this important gene as a target of further investigation for the pathogenesis of COPD and as a potential target of therapy.

## Abbreviations

COPD: chronic obstructive pulmonary disease; EPHX1: microsomal epoxide hydrolase; FEV1: forced expiratory volume in one second; FVC: forced vital capacity; PPARG: peroxisome proliferator-activated receptor gamma

## Competing interests

LN has no conflicts of interests (COIs) to disclose. AP has no COIs to disclose. SP has no COIs to disclose. ECs has no COIs to disclose. BS has no COIs to disclose. BD has no COIs to disclose. IS has no COIs to disclose. IK is director of Pfizer and holder of Pfizer stock. LT has no COIs to disclose.

## Authors' contributions

LN is an International Scholar of HHMI and holds a Wellcome Trust Senior Research Fellowship in Biomedical Sciences, he planned and directed the study. AP performed data analyses and prepared the manuscript. SP performed the genotyping measurements and RT-QPCR analyses and prepared figures and tables. ECs organized patient recruitment and sample collection. BS organized sample preparation. BD performed IHC staining. IS performed data analyses. IK and LT participated in the planning and the design of the study and helped prepare the manuscript.

All authors have read and approved the final version of the manuscript.

## Pre-publication history

The pre-publication history for this paper can be accessed here:

http://www.biomedcentral.com/1471-2350/11/152/prepub

## Supplementary Material

Additional File 1 **Table S1. Characteristics of tested SNP**. Characteristics, NCBI reference numbers and ABI assays code of examined single nucleotide polymorphisms. Click here for file
