# Supplementary material for: Analyses of association between PPAR gamma and EPHX1 polymorphisms and susceptibility to COPD in a Hungarian cohort, a case-control study
Source: BMC Med Genet. 2010 Nov 2;11:152. doi: 10.1186/1471-2350-11-152 (PMC2988760; doi:10.1186/1471-2350-11-152)
Supplement: Additional File 1 — Table S1. Characteristics of tested SNP. Characteristics, NCBI reference numbers and ABI assays code of examined single nucleotide polymorphisms. [file 1471-2350-11-152-S1.DOC]

| Gene Symbol | NCBI SNP reference number | SNP Type | Alleles and characteristics | ABI TaqMan genotyping assay ID |
| --- | --- | --- | --- | --- |
| EPHX1 | rs1051740 | Exon 3 Tyr113His; T/C | His; "slow" allele | C_14938_30 |
|  | rs2234922 | Exon 4 His139Arg; A/G | Arg; "fast" allele | C_11638783_30 |
| PPARG | rs1801282 | Exon B, Pro12Ala; C/G | Non-synonymus polymorphism | C_26856791_20 |
|  | rs3856806 | Exon 6, His447His; C/T | Synonymus polymorphism | C_11922961_30 |
|  | rs1800571 | Exon 3, Pro115Gln; C/A | Non-synonymus polymorphism | C_8756581_20 |

Additional Table 1. Characteristics of examined SNPs
